# Effect of preoperative contralateral foramen stenosis on contralateral root symptoms after unilateral transforaminal lumbar interbody fusion: a ambispective cohort study

**DOI:** 10.1186/s12891-023-06381-2

**Published:** 2023-04-14

**Authors:** Wenjie Lu, Lingqiao Wu, Yunlin Chen, Xudong Hu, Chaoyue Ruan, Yang Wang, Weihu Ma, Weiyu Jiang

**Affiliations:** 1 Zhejiang Chinese Medical University, Hangzhou, 310053 Zhejiang China; 2 Department of Spinal Surgery, Ningbo Sixth Hospital, Ningbo, 315040 Zhejiang China

**Keywords:** Unilateral, Transforaminal interbody fusion, Degree of contralateral intervertebral foramen stenosis, Contralateral root symptoms, Incidence, Relevance

## Abstract

**Purpose:**

To evaluate the correlation between the degree of preoperative contralateral foraminal stenosis(CFS) and the incidence of contralateral root symptoms after unilateral transforaminal lumbar interbody fusion(TLIF) and to evaluate the appropriate candidate of preventive decompression according to the degree of preoperative contralateral foraminal stenosis.

**Methods:**

An ambispective cohort study was conducted to investigate the incidence of contralateral root symptoms after unilateral transforaminal lumbar interbody fusion (TLIF) and the effectiveness of preventive decompression. A total of 411 patients were included in the study, all of whom met the inclusion and exclusion criteria and underwent surgery at the Department of Spinal Surgery, Ningbo Sixth Hospital, between January 2017 and February 2021. The study was divided into two groups: retrospective cohort study A and prospective cohort study B. The 187 patients included in study A from January 2017 to January 2019 did not receive preventive decompression. They were divided into four groups based on the degree of preoperative contralateral intervertebral foramen stenosis: no stenosis group A1, mild stenosis group A2, moderate stenosis group A3, and severe stenosis group A4. A Spearman rank correlation analysis was used to evaluate the correlation between the preoperative contralateral foramen stenosis degree and the incidence of contralateral root symptoms after unilateral TLIF. From February 2019 to February 2021, 224 patients were included in the prospective cohort group B. The decision to perform preventive decompression during the operation was based on the degree of preoperative contralateral foramen stenosis. Severe intervertebral foramen stenosis was treated with preventive decompression as group B1, while the rest were not treated with preventive decompression as group B2. The baseline data, surgical-related indicators, the incidence of contralateral root symptoms, clinical efficacy, imaging results, and other complications were compared between group A4 and group B1.

**Results:**

All 411 patients completed the operation and were followed up for an average of 13.5 ± 2.8 months. In the retrospective study, there was no significant difference in baseline data among the four groups (P > 0.05). The incidence of postoperative contralateral root symptoms increased gradually, and a weak positive correlation was found between the degree of preoperative intervertebral foramen stenosis and the incidence of postoperative root symptoms (rs = 0.304, P < 0.001). In the prospective study, there was no significant difference in baseline data between the two groups. The operation time and blood loss in group A4 were less than those in group B1 (P < 0.05). The incidence of contralateral root symptoms in group A4 was higher than that in group B1 (P = 0.003). However, there was no significant difference in leg VAS score and ODI index between the two groups at 3 months after the operation (P > 0.05). There was no significant difference in cage position, intervertebral fusion rate, and lumbar stability between the two groups (P > 0.05). No incisional infection occurred after the operation. No pedicle screw loosening, displacement, fracture, or interbody fusion cage displacement occurred during follow-up.

**Conclusion:**

This study found a weak positive correlation between the degree of preoperative contralateral foramen stenosis and the incidence of contralateral root symptoms after unilateral TLIF. Intraoperative preventive decompression of the contralateral side may prolong the operation time and increase intraoperative blood loss to some extent. However, when the contralateral intervertebral foramen stenosis reaches the severe level, it is recommended to perform preventive decompression during the operation. This approach can reduce the incidence of postoperative contralateral root symptoms while ensuring clinical efficacy.

## Background

Transforaminal lumbar interbody fusion (TLIF) is a refined surgical technique that has evolved from posterior lumbar interbody fusion (PLIF). It was first introduced by Blume and Rojas in the early 1980s and has now become a classic surgical method for treating lumbar degenerative diseases, gradually replacing PLIF [1]. TLIF, using a unilateral transforaminal approach, can avoid excessive traction of the dural sac, nerve roots, and lumbar and back muscles, while achieving bilateral decompression by preserving the contralateral lamina and facet joints. This technique has a minimal impact on the mechanical structure of the spine’s posterior column, while retaining bone structures such as the pedicle and lamina, which increases the stability between the adjacent vertebral bodies [2]. Long-term clinical studies have shown that TLIF has satisfactory clinical efficacy. However, it is not without complications, and contralateral root symptoms after unilateral TLIF are a common complication that may affect the overall efficacy of the surgery [3, 4]. Previous studies by domestic and international scholars have identified preoperative contralateral intervertebral foramen stenosis, large lumbar sagittal plane mobility, and intraoperative fusion device position to one side as important risk factors for the development of contralateral root symptoms after unilateral TLIF [5, 6]. However, these studies have several limitations, including a lack of quantification of research results and limited clinical significance. To address these limitations, we conducted a two-way cohort study. Firstly, we retrospectively reviewed cases of unilateral TLIF in our hospital from January 2017 to January 2019, using Spearman rank correlation analysis to determine the correlation between the degree of preoperative intervertebral foramen stenosis and the incidence of contralateral root symptoms after unilateral TLIF. Secondly, we conducted a prospective study on patients who underwent unilateral TLIF in our hospital from February 2019 to February 2021, comparing the effects of intraoperative prophylactic decompression on postoperative clinical efficacy, imaging results, and the incidence of contralateral radicular symptoms in patients with preoperative contralateral severe foramen stenosis. Our study aimed to determine the appropriate candidate of prophylactic decompression.

## Information and methods

### Inclusion and exclusion criteria

The inclusion criteria were low back pain with unilateral nerve root symptoms, surgery for unilateral TLIF, the surgical segment between L3 and S1, lesions involving four segments below and we excluded patients who had bilateral nerve root symptoms or severe systemic diseases like lumbar trauma, tumors, severe osteoporosis, birth defects or surgeries involving more than four segments, non-unilateral TLIF, or those lacking long-term complete follow-up and clinical data.

### General information and case subgroups

We utilized the grading system proposed by [7]. to evaluate the degree of intervertebral foramen stenosis. On sagittal T1-weighted images, the degree of stenosis was graded based on the morphology of the intervertebral foramen epidural fat. A grade of 0 indicated no stenosis, grade 1 indicated mild stenosis with transverse or longitudinal reduction of the perineural fat space, grade 2 indicated moderate stenosis characterized by decreased transverse and longitudinal fat space without neuromorphological changes, and grade 3 indicated severe stenosis with morphological changes or destruction of nerve roots. This study was approved by the Ethics Committee of Ningbo Sixth Hospital. All methods were in accordance with the Helsinki Declaration and its contemporary amendments. There is no information or marker related to patient identity in any of the data. The informed consent was obtained from all subjects and/or their legal guardian(s).

A total of 187 patients with lumbar degenerative disease who underwent unilateral TLIF were included in the retrospective study. They were divided into four groups according to the degree of preoperative contralateral intervertebral foramen stenosis: no stenosis group A1 (42 cases, 25 males and 17 females, aged 55.8 ± 12.7 years), mild stenosis group A2 (57 cases, 32 males and 25 females, aged 53.5 ± 11.8 years), moderate stenosis group A3 (51 cases, 28 males and 23 females, aged 56.1 ± 9.4 years), and severe stenosis group A4 (37 cases, 21 males and 16 females, aged 58.9 ± 16.2 years).

A total of 224 patients with lumbar degenerative disease who underwent unilateral TLIF were included in the prospective study. Patients with severe stenosis of the contralateral intervertebral foramen before the operation and preventive decompression during the operation were classified as the decompression group B1 (56 cases, 30 males and 26 females, aged 55.2 ± 10.2 years). Patients with non-severe stenosis before the operation and without preventive decompression during the operation were classified as the non-decompression group B2 (168 cases, 96 males and 72 females, aged 51.8 ± 8.7 years).

In this study, the subjects were divided into a non-decompression group A4 and a decompression group B1, and followed up for 13.5 ± 2.8 months. The incidence in the decompression group was approximately 0.3, the incidence in the non-decompression group was approximately 0.27, and the relative risk was approximately 0.11, based on previous studies [8, 9]. Considering bilateral α = 0.05 and test efficacy of 80%, a minimum of 31 subjects were required in each group. Given the small sample size involved in this study, we included all cases that met the inclusion criteria to reduce the error caused by the small sample size to a certain extent.

### Operation and postoperative treatment

All patients underwent conservative treatments for more than three months before surgery, including bed rest, physical therapy, low back muscle function exercise, and drug therapy consisting of neurotrophic drugs, non-steroidal anti-inflammatory drugs, hormones, and muscle relaxants. Unilateral transforaminal lumbar interbody fusion (TLIF) was performed by two senior surgeons from the same institution (W-Y.J. and W-H.M.). The patient was placed in the prone position under general anesthesia, and a longitudinal incision was made on the symptomatic side of the lumbar spine to expose the interspace between the erector spinae and multifidus muscles. The interspace was gradually dilated to insert a Quadrant working channel, revealing part of the lamina and articular processes. Part of the lamina and articular processes were removed, with the articular processes not exceeding one-third of the medial aspect. The nerve roots, dura mater, and free yellow ligament were then exposed. If contralateral preventive decompression was performed, the surgical table was tilted approximately 30 degrees to the opposite side, and a burr was used to remove the bone at the base of the upper vertebral spinous process on the dorsal side of the yellow ligament, followed by gradual removal of the inner layer of the contralateral lamina and medial bony structure of the articular processes. The yellow ligament was scraped off, and bilateral nerve roots and dura mater were decompressed before the surgical table was returned to its original position. The intervertebral nucleus and endplate cartilage were removed, and autologous bone chips were implanted between the vertebral bodies, followed by insertion of a vertebral interbody fusion cage filled with some autologous bone chips. Under the guidance of a C-arm X-ray machine, screws were placed on the contralateral side through the lamina and articular processes. Two appropriate pedicle screws were inserted on the side of the surgical incision, followed by installation of titanium rods, appropriate compression between the pedicle screws, tightening of the tail cap, placement of a drainage tube, irrigation and closure of the incision, and covering with sterile dressings.

After the recovery from anesthesia, patients were administered NSAIDs and methylprednisolone to relieve nerve root pain and inflammation (On the first day after surgery, a dose of 500 mg methylprednisolone was dissolved in 500ml of 0.5% physiological saline and infused intravenously in 1 h. On the second day, a dose of 160 mg methylprednisolone was dissolved in 500ml of 0.5% physiological saline and infused intravenously in 1 h. Finally, on the third day, a dose of 80 mg methylprednisolone was dissolved in 500ml of 0.5% physiological saline and infused intravenously in 1 h). Elastic socks and an arteriovenous foot pump were used to prevent deep vein thrombosis, starting 6 h after the operation. Once the drainage tube was removed, all patients began walking under brace protection. If patients experienced postoperative contralateral nerve root symptoms that did not improve after NSAID administration, a single 1% lidocaine injection of 0.5ml was administered. If the root symptoms improved at least 80% after injection, it indicated nerve root lesion and further lumbar MRI was required.

### Evaluation indicators

Radiographs of the lumbar spine, including anteroposterior, lateral, and dynamic images, were taken at four different intervals post-operation: 1 week, 3 months, 6 months, and 12 months. Additionally, a lumbar CT scan was conducted 1 week after the operation to assess the position of pedicle screws and the cage. Twelve months post-operation, lumbar CT scans and three-dimensional reconstructions were performed to observe whether the pedicle screws had become loose or broken, whether the fusion cage had subsided or displaced, and whether intervertebral fusion had occurred. For patients exhibiting contralateral root symptoms, an additional lumbar MRI examination was required, and a nerve root block was performed, if necessary, to identify the root cause. The screw position evaluation referred to the standard established by Liu Qingyu et al. [10]. The presence of postoperative hematoma was evaluated by comparing the signal shadow on the acute MRI T2WI image to an equal or slightly lower signal shadow of progressive enlargement.

1. Clinical efficacy evaluation.

Visual Analog Scale

At 72 h after the operation, leg radiating pain was scored and compared using the visual analog scale (VAS). Patients were asked to indicate the degree of pain they experienced on a 10-point horizontal ruler closest to the level of pain they marked, thereby allowing for quantification of the degree of pain: 0 points represented no pain, while 1–3 points, 4–6 points, and 7–10 points represented mild pain, moderate pain, and severe pain, respectively.

Oswestry Disability Index

To evaluate the changes in lumbar function before and at the final follow-up, the Oswestry Disability Index (ODI) questionnaire was utilized [11, 12]. The ODI questionnaire consists of 10 aspects, including the degree of pain, daily self-care ability, ability to lift objects, walking, sitting, standing, sleeping, sexual function, social activities, and ability to travel. Each aspect had six options, and each option was assigned a score ranging from 0 to 5 points. The scoring method was calculated by dividing the actual score by 50 (the highest possible score) and multiplying the result by 100%. Higher scores indicate more serious lumbar dysfunction.

2. Imaging evaluation.

Lumbar stability

**Fig. 1:**
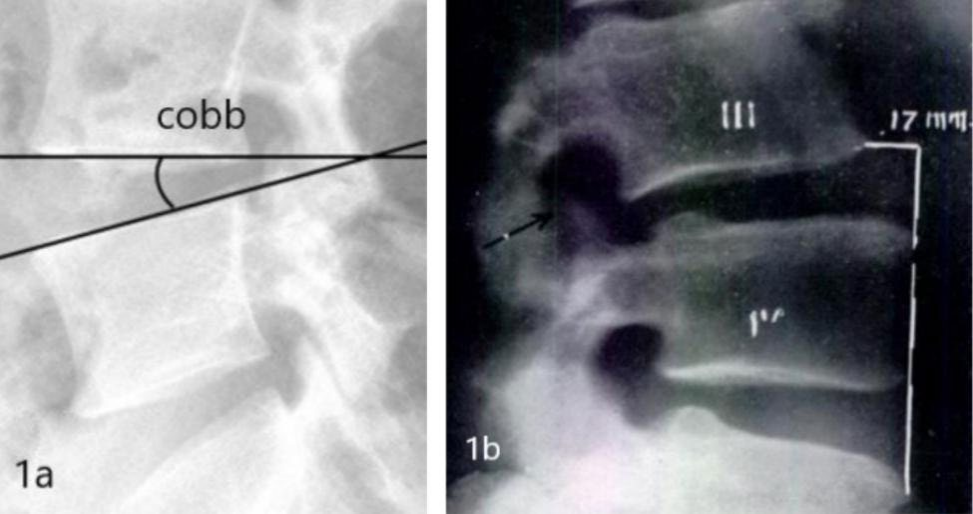
**1a.** Cobb angle between vertebrae: the angle between the upper and lower endplates of the same segment before and after operation; **1b.** vertebral slip distance: changes in relative slip distance before and after surgery

The imaging diagnostic criteria for lumbar instability were lumbar lateral X-ray film and flexion and extension dynamic X-ray film comparison in the same segment, lumbar intervertebral Cobb angle change > 11°, or vertebral slip distance change > 4 mm [13–15]( Fig. 1).

Intervertebral fusion rate

At the 12-month follow-up, two groups of patients who had undergone intervertebral bone graft fusion (Groups A and B) were evaluated using lumbar CT scan and three-dimensional reconstruction. Intervertebral fusion was assessed using the modified Brantigan score [16], which assigns points based on the degree of fusion observed: 4 points for complete fusion and good shaping, 3 points for good fusion with a small amount of transparent line, 2 points for the upper and lower parts being 50% connected but with many bright lines still present, 1 point for the upper and lower parts not being connected but with an increase in bone mass compared to the immediate bone graft after the operation, and 0 points for the upper and lower parts not being connected, with height loss and bone graft absorption. A score of ≥ 3 points indicates fusion.

**Fig. 2:**
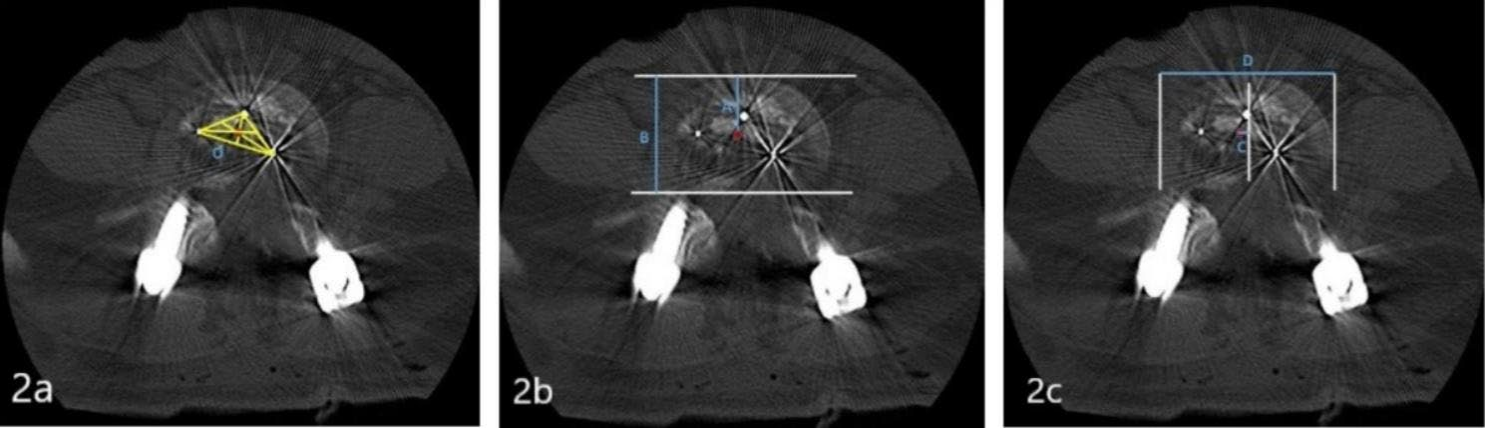
**2a**.marked the center position of the fusion cage, that was, the center of gravity of the triangle formed by the three marker points of the cage; **2b.**The distance between the center of the cage and the front edge of the vertebral body was A, and the sagittal diameter of the intervertebral disc was B. A/B represented the cage’s sagittal position; **2c.**The distance from the center of the cage to the bisector of the coronal plane of the intervertebral disc was C, and C had positive and negative points. When the center of the cage was biased toward the side of the cage, it was negative. When the center of the cage was biased toward the other side, it was positive. D was the length of the intervertebral disc’s left and right diameters, and C/D represented the cage’s coronal position

The position of the fusion cage (both sagittal and coronal) was measured on the cross-section of the CT scan. The central position of the cage was marked as the center of gravity of the triangle formed by the three marker points of the cage (Fig. 2a). The distance between the center of the cage and the front edge of the vertebral body was measured as A, and the sagittal diameter of the intervertebral disc was measured as B. The ratio of A to B represented the sagittal position of the cage (Fig. 2b). The distance from the center of the cage to the bisector of the coronal plane of the intervertebral disc was measured as C, which could be positive or negative depending on the position of the cage relative to the midline. The length of the intervertebral disc’s left and right diameters was represented as D, and the ratio of C to D indicated the coronal position of the cage (Fig. 2c) [17].

3. Evaluation of complications.

Observed and compared were the complications of group A and group B, including intraoperative dural injury, nerve injury, postoperative screw loosening, fracture, intervertebral fusion cage displacement, infection, and others.

All the above parameters were measured by a spinal surgeon with over ten years of experience in spinal surgery and a musculoskeletal system specialist radiologist. The final results were measured using the average of the two measurements.

### Statistical methods

Statistical analysis was performed using SPSS 25.0. The measurement data conforming to a normal distribution were expressed as mean ± standard deviation (X ± S), and an independent sample t-test was used. The measurement data not conforming to the normal distribution were expressed as median (minimum to maximum), and the Mann-Whitney U test was used. For count data, the Pearson chi-square (X^2^) test was used when the minimum theoretical frequency was ≥ 5, and the Fisher exact test was used when the minimum theoretical frequency was < 5. One-Way ANOVA was used to compare the mean difference of each index in different periods, and the least significant difference (LSD) method was used to compare between groups. The Spearman rank correlation analysis was used to evaluate the correlation between the degree of preoperative contralateral intervertebral foramen stenosis and the incidence of postoperative contralateral root symptoms. A p-value less than 0.05 was considered statistically significant.

## Results

All 411 patients underwent successful surgery and were followed up for an average of 13.5 ± 2.8 months. From this retrospective study, 187 cases were included based on the predetermined inclusion and exclusion criteria. Of these cases, 16 patients experienced contralateral root symptoms after the surgery. The patients were divided into four groups based on the degree of preoperative contralateral intervertebral foramen stenosis, and baseline data, including gender, age, and preoperative diagnosis, were not significantly different among the groups (P > 0.05) (Table 1). The incidence of contralateral root symptoms increased progressively across the four groups (0%, 3.51%, 7.84%, 27.03%), with the incidence in group A4 being significantly higher than those in groups A1, A2, and A3 (P < 0.001) (Table 2). Spearman rank correlation analysis revealed a weak positive correlation between the degree of preoperative intervertebral foramen stenosis and the incidence of postoperative root symptoms (rs = 0.304, P < 0.001).

**Table 1:** A1, A2, A3 and A4 four groups of baseline data comparison

Data/Group	Group A1	Group A2	Group A3	Group A4	P Value
Number	42	57	51	37	—
Gender (male/female)	25/17	32/25	28/23	21/16	0.976
Age (years)	51.8 ± 12.7	53.5 ± 11.8	52.1 ± 11.4	54.9 ± 13.2	0.533
Diagnosis (SS/LS/LDH)	2026/7/9	1930/12/15	2029/11/11	2024/6/7	0.93
Preoperative CFS degree	No stenosis	Mild stenosis	Moderate stenosis	Severe stenosis	—

Note: P < 0.05 was considered statistically significant. Spinal stenosis-SS; Lumbar spondylolisthesis-LS; Lumbar disc herniation-LDH

**Table 2:** Comparison of the incidence of contralateral root symptoms after unilateral TLIF in four groups

Data/Group	Group A1	Group A2	Group A3	Group A4	P Value
Number	42	57	51	37	—
Number of patients	0	2	4	10	—
Incidence of PRS(%)	0(0/42)	3.51(2/57)	7.84(4/51)	27.03(10/37)	＜0.001

Note: P < 0.05 was considered statistically significant. Postoperative root symptoms-PRS

**Fig. 3:**
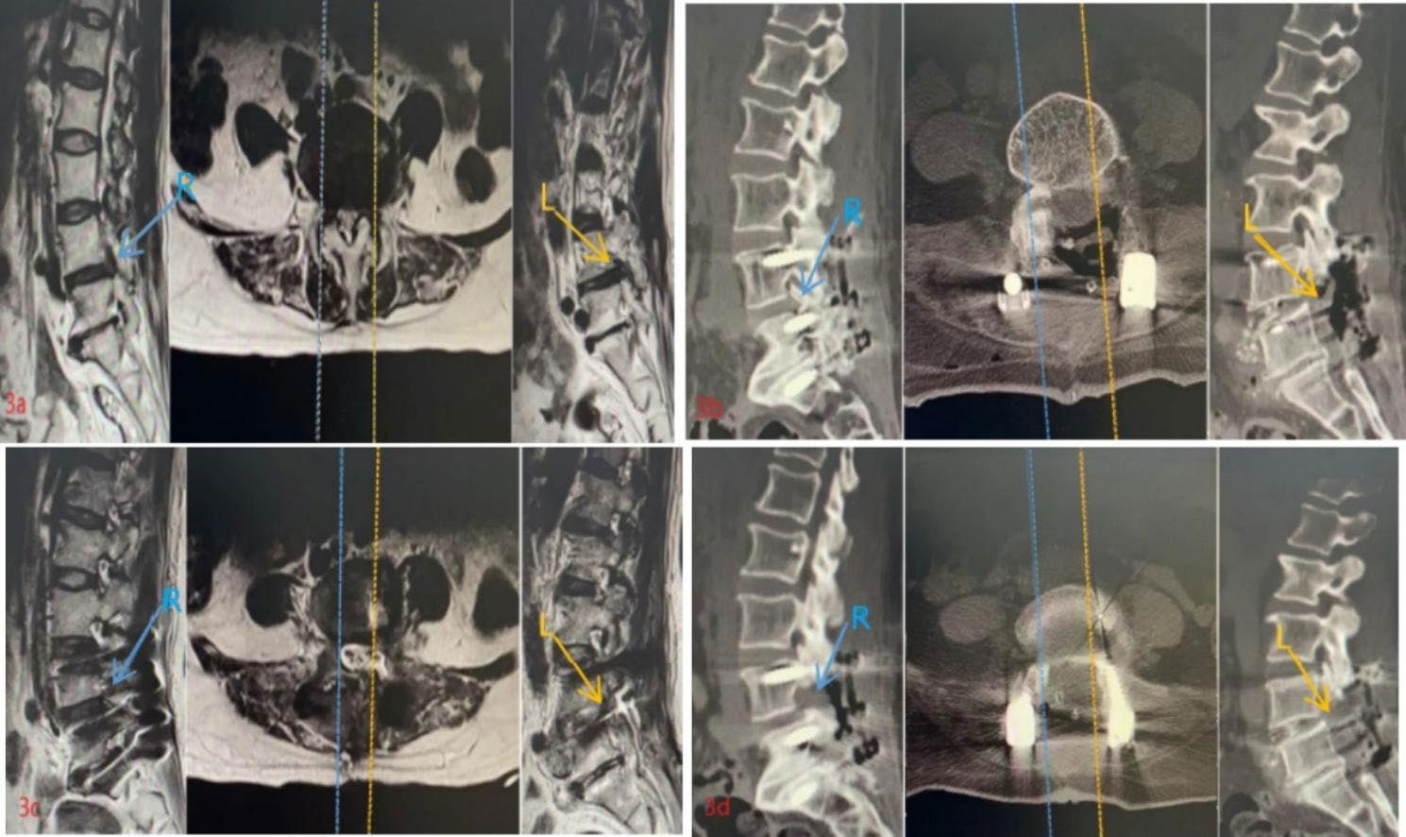
A 56-year-old female patient was admitted to the hospital due to low back pain with left lower limb pain and numbness for 5 years, which had been aggravated for 1 week. Lumbar decompression and interbody fusion were performed through the left intervertebral foramen of L4 and 5, and no preventive decompression was performed on the right side. On the fifth day after the operation, the patient had grade 2 right ankle dorsiflexion muscle strength, grade 1 right hallux dorsiflexion muscle strength, and shallow sensation of the right hallux toe skin. The symptoms were not significantly improved after dehydration and conservative treatment. Subsequently, lumbar CT and MRI examinations were performed. Considering the dislocation of the right superior articular process of the lower vertebral body, emergency nerve root release was performed, and postoperative recovery was acceptable. **3a**, L4/5 bilateral intervertebral foramen severe stenosis; **3b,3c**, Lumbar decompression and interbody fusion was performed through the left intervertebral foramen of L4/5, and no preventive decompression was performed on the right side during the operation; **3d**, After the operation, the symptoms of right intervertebral foramen stenosis occurred, and nerve root release was performed. The right inferior articular process of L4 and the right superior articular process of L5 were removed with bite forceps

**Fig. 4:**
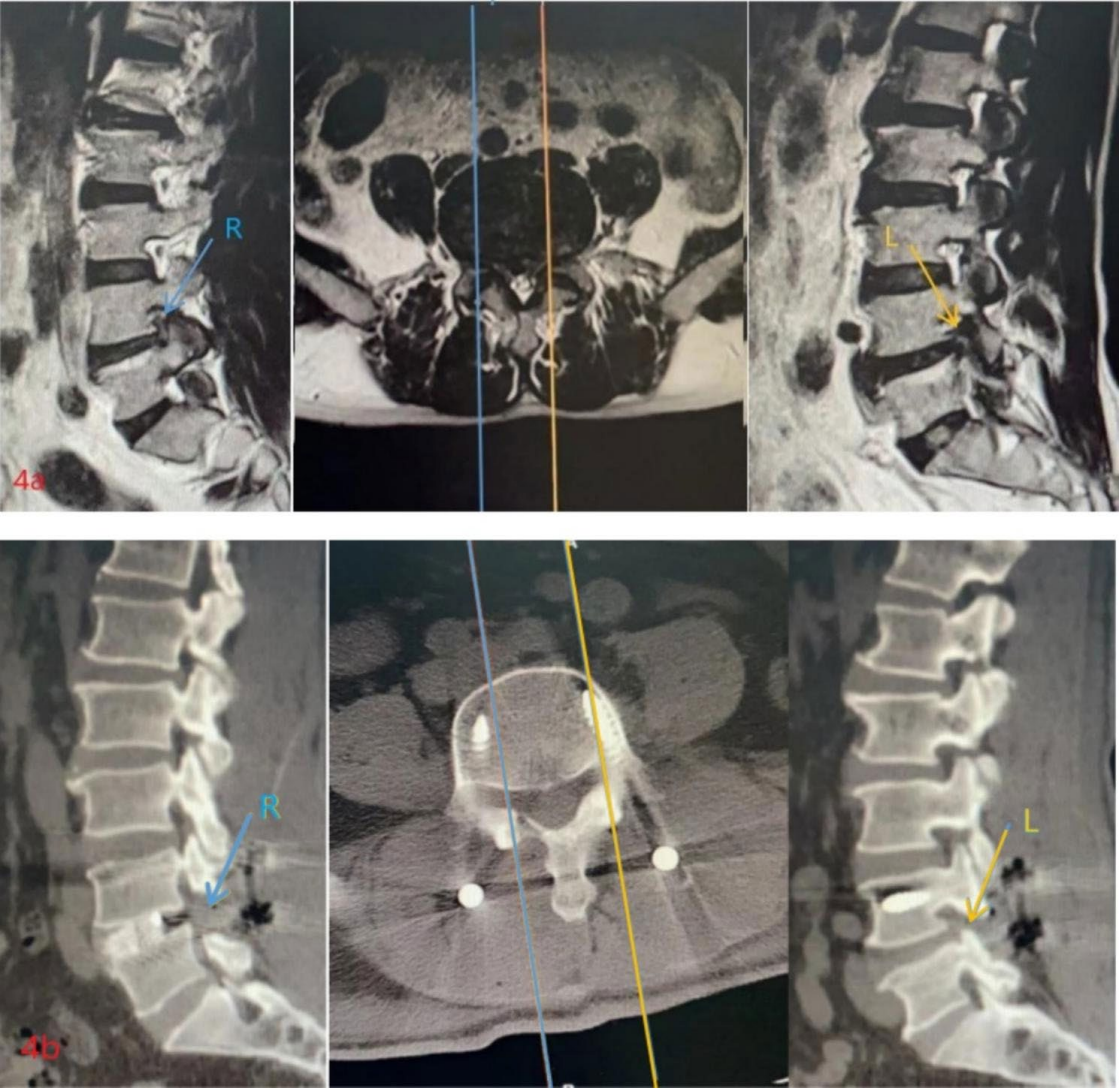
A 60-year-old male patient with low back pain and right lower limb pain and numbness was hospitalized for one month. Lumbar decompression and interbody fusion was performed through the right intervertebral foramen of L4/5. The cortical bone of L4/5 left facet joint and transverse process were removed during operation. during the operation, and preventive decompression was performed. The operation was successful, the postoperative recovery was good, and no contralateral root symptoms occurred. **4a**, Preoperative MRI showed severe stenosis of the L4/5 bilateral intervertebral foramen. **4b**, The right lamina and facet joint of L4/5 were removed. The cortical bone of L4/5 left facet joint and transverse process were removed

In this prospective study, a total of 224 patients were included and divided into two groups based on whether contralateral preventive decompression was performed during the operation. Group B1 underwent contralateral preventive decompression, while group B2 did not. There was no significant difference in baseline data, including gender, age, and preoperative diagnosis, between group A4 and group B1(Table 3). The operation time was significantly shorter in group A4 than in group B1, while the intraoperative blood loss was significantly lower in group A4 than in group B1. Of the 37 patients in group A4, 10 (27.03%) developed contralateral root symptoms, while only 3 (5.36%) of the 56 patients in group B1 had similar symptoms. The difference in incidence between the two groups was statistically significant. However, there was no significant difference in leg VAS score and ODI index between the two groups three months after the operation, and lumbar stability was not significantly different between the two groups before and after the operation. At the one-year follow-up, the interbody fusion rate was high in both groups, with no significant difference between the two. No complications such as incisional infection, pedicle screw loosening, displacement, fracture, or interbody fusion cage displacement occurred during the follow-up (Tables 4 and 5). Typical cases are shown in Figs. 3 and 4. The findings of this study provide important insights into the efficacy and safety of contralateral preventive decompression in patients undergoing lumbar spinal stenosis surgery.

**Table 3:** Comparison of baseline data between group A4 and group B1

Data/Group	Group A4	Group B1	P Value
Number	37	56	—
Gender (male/female)	21/16	30/26	0.763
Age (years)	54.9 ± 13.2	55.2 ± 10.2	0.902
Diagnosis (SS/LS/LDH)	2024/6/7	1931/10/15	0.619
Preoperative CFS degree	Severe stenosis	Severe stenosis	—

Note: P < 0.05 was considered statistically significant. Spinal stenosis: SS; Lumbar spondylolisthesis: LS; Lumbar disc herniation: LDH

**Table 4:** Comparison of surgical indexes, cage position, the incidence of root symptoms, and the intervertebral fusion rate between group A4 and group B1

Data/Group		Group A4	Group B1	P Value
Surgical index(min/ml)				
	Operation time	78.3 ± 16.1	95.2 ± 19.5	＜0.001
	Blood loss	118.3 ± 32.2	132.3 ± 22.5	0.015
Fusion cage position(%)				
	Sagittal position	44.5 ± 6.8	42.2 ± 5.7	0.081
	Coronal position	-6.5 ± 7.1	-5.2 ± 7.3	0.398
Incidence of PRS(%)		27.03(10/37)	5.36(3/56)	0.003
Fusion rate(%)		97.3(36/37)	96.4(54/56)	0.653

Note: P < 0.05 was considered statistically significant. Postoperative root symptoms-PRS

**Table 5:** Comparison of clinical effects between group A4 and group B1 (x ± s)

Data/Group		Group A4	Group B1	P Value
VAS score(leg)				
	preoperative	7.46 ± 1.34	8.01 ± 1.55	0.081
	3 months after operation	2.16 ± 1.24	1.78 ± 0.94	0.097
	P value	＜0.001	＜0.001	
ODI index				
	preoperative	57.46 ± 6.34	59.38 ± 8.45	0.961
	3 months after operation	11.20 ± 5.18	12.36 ± 4.71	0.267
	P value	＜0.001	＜0.001	

Note: P < 0.05 was considered statistically significant

## Typical cases

## Discussion

Transforaminal lumbar interbody fusion (TLIF) is a surgical technique that enables access to the intervertebral space via a unilateral intervertebral foramen, allowing for bilateral decompression and providing sufficient biomechanical stability to facilitate intervertebral fusion. TLIF is widely utilized in clinical practice and has demonstrated excellent therapeutic outcomes. However, the use of a unilateral approach often results in nerve root symptoms caused by contralateral foraminal stenosis (CFS). Studies have indicated that the incidence of contralateral root symptoms after minimally invasive surgery (MIS-TLIF) ranges from 1.9 to 6.9% in open TLIF and 3.0% in MIS-TLIF. The incidence of endoscopically assisted MIS-TLIF is relatively high at 8.5% [8, 9]. Given the relatively high incidence of postoperative contralateral root symptoms as a significant complication, clinicians cannot overlook this issue.

### Analysis of the research status of contralateral root symptoms after unilateral TLIF

Since Hunt et al. first reported a case of contralateral root symptoms after unilateral TLIF in 2007, several researchers have conducted extensive investigations into its incidence, causes, and risk factors [18]. However, previous studies have several limitations. For example, the data obtained in Hunt’s study were not measured at the same sagittal plane of CT, which raises concerns about their reliability, and their clinical significance is limited [19, 20]. In Yang Y’s study, CFA was measured on the sagittal plane of lumbar CT. However, considering the anatomical relationship, CFA measured on the sagittal plane of lumbar CT may not accurately depict the relationship between the nerve root and the intervertebral foramen [21–23].

### Relationship between contralateral foramen stenosis and contralateral radicular symptoms after unilateral TLIF

Studies have suggested that TLIF can alleviate contralateral foraminal stenosis through indirect decompression. However, CFA did not always increase clinically as anticipated, especially in patients with preexisting contralateral foraminal stenosis who may develop new neurological symptoms [24–26]. When the position of the fusion cage is biased towards the decompression side, it can lead to an imbalanced internal stress distribution of the intervertebral space and tilt the intervertebral space, resulting in increased intervertebral foramen stenosis on the non-decompression side, causing root symptoms. Additionally, unilateral TLIF only removes part of the facet joints on one side, resulting in less damage to the posterior structure of the lumbar spine. The anterior bone graft in the intervertebral space may cause the position of the fusion device to deviate from the middle and posterior sides of the intervertebral space. As a result, the hinge fulcrum moves backward, and the force arm becomes smaller. To restore lumbar lordosis, greater stresses are frequently required, which may cause the upper articular process of the lower vertebral body to move upward or ventrally, leading to increased stenosis of the intervertebral foramen on the uncompressed side and root symptoms [27]. Therefore, patients with preoperative contralateral foramen stenosis are more likely to develop root symptoms after surgery. In this study, there was no significant difference in the position of the fusion cage between group A4 and group B1 (P > 0.05), indicating that the position of the fusion cage was not a contributing factor affecting the incidence of contralateral root symptoms after unilateral TLIF.

#### Analysis of research results

In the retrospective study, 37 patients in the A4 group with preoperative severe stenosis of the contralateral intervertebral foramen underwent surgery. Following the operation, 10 cases of contralateral root symptoms occurred, resulting in an incidence rate of 27.03%. As the degree of preoperative contralateral intervertebral foramen stenosis worsened, the incidence of postoperative contralateral root symptoms gradually increased in groups A1, A2, A3, and A4. Spearman rank correlation analysis indicated a weak positive correlation between the degree of preoperative intervertebral foramen stenosis and the incidence of postoperative root symptoms (rs = 0.304, P < 0.001), consistent with previous studies [22, 23]. However, the author noted that this correlation was weak, and a larger, more comprehensive study would be necessary to consider additional risk factors and improve the results.

In the prospective study, 56 patients in group B1 had severe stenosis of the contralateral intervertebral foramen before the operation, with 3 cases of contralateral root symptoms occurring afterward. The incidence rate was 5.36%, which was statistically different from that of group A4 (27.03%) (P = 0.003), suggesting that intraoperative contralateral preventive decompression could reduce the incidence of postoperative contralateral root symptoms to a certain extent. The operation time and intraoperative blood loss in group A4 were less than those in group B1, and the difference was statistically significant (P < 0.05). Thus, preventive decompression of the contralateral side might prolong the operation time and increase intraoperative blood loss. Follow-up at 3 months revealed no significant difference in leg VAS score and ODI index between the two groups (P < 0.05), indicating that intraoperative contralateral preventive decompression did not affect postoperative clinical efficacy. Cross-sectional CT scans taken 1 week after the operation showed no significant difference in the position of the fusion cage between the two groups, while lumbar lateral and dynamic radiographs indicated no significant difference in lumbar stability between the two groups before and after the operation. At 1 year follow-up, the interbody fusion rate was 97.3% in group A4 and 98.8% in group B1, and there was no significant difference between the two groups (P = 0.653), suggesting that intraoperative contralateral preventive decompression did not affect the postoperative interbody fusion rate and lumbar stability. Moreover, there was no incisional infection in the 93 patients across the two groups, and no loosening, displacement, fracture of the pedicle screw, or displacement of the intervertebral fusion cage occurred during follow-up, indicating that intraoperative contralateral preventive decompression did not increase the incidence of postoperative complications.

Nevertheless, the study has some limitations. Although the relationship between the degree of foraminal stenosis and contralateral radicular symptoms after unilateral TLIF was analyzed, other risk factors occurring during and after the operation were not included. Additionally, this study defined severe stenosis as the standard of contralateral preventive decompression during the operation, which is only based on the author’s long-term clinical experience, without a perfect theoretical basis. Therefore, further multi-center high-quality research with a larger sample size and a more robust theoretical foundation is required to improve the findings.

## Conclusion

The retrospective and prospective studies conducted on unilateral TLIF have shed light on several important aspects of the surgery. The studies have revealed that there exists a weak positive correlation between the degree of preoperative contralateral foramen stenosis and the incidence of contralateral root symptoms after the surgery. Moreover, intraoperative preventive decompression of the contralateral side is associated with a prolonged operation time and increased intraoperative blood loss. However, when the contralateral intervertebral foramen reaches severe stenosis, preventive decompression during the operation is recommended to reduce the incidence of postoperative contralateral root symptoms without affecting the postoperative clinical efficacy. Therefore, the studies emphasize the importance of considering the severity of contralateral foramen stenosis and making informed decisions regarding preventive decompression during unilateral TLIF.

## Data Availability

The datasets used and/or analysed during the current study available from the corresponding author on reasonable request.
